# A Simple Electrochemical Method for the Rapid Estimation of Antioxidant Potentials of Some Selected Medicinal Plants

**Published:** 2012

**Authors:** Salimeh Amidi, Faraz Mojab, Abdolmajid Bayandori Moghaddam, Kimia Tabib, Farzad Kobarfard

**Affiliations:** a *Department of Medicinal Chemistry, School of Pharmacy, Shahid Beheshti University of Medical Sciences, Tehran, Iran.*; b *Department of Pharmacognosy, School of Pharmacy, Shahid Beheshti University of Medical Sciences, Tehran, Iran.*; c *Nanotechnology Research Center, Faculty of Pharmacy, Tehran University of Medical Sciences, Tehran, Iran. *; d *Phytochemistry Research Center, Shahid Beheshti University of Medical Sciences.*

**Keywords:** Cyclic voltammetry, Antioxidant, Plant extracts, Electrochemical

## Abstract

Clinical and Epidemiological studies have shown that a diet rich in fruits and vegetables is associated with a decreased risk of cardiovascular diseases, cancers and other related disorders. These beneficial health effects have been attributed in part to the presence of antioxidants in dietary plants. Therefore screening for antioxidant properties of plant extracts has been one of the interests of scientists in this field.

Different screening methods have been reported for the evaluation of antioxidant properties of plant extracts in the literature. In the present research a rapid screening method has been introduced based on cyclic voltammetry for antioxidant screening of some selected medicinal plant extracts.

Cyclic Voltammetry of methanolic extracts of seven medicinal plants: *Buxus hyrcana, Rumex crispus, Achillea millefolium, Zataria* *multiflora, Ginkgo biloba, Lippia citriodora* and *Heptaptera anisoptera *was carried out at different scan rates.

Based on the interpretation of voltammograms, *Rumex crispus*, *Achillea millefolium* and *Ginkgo biloba* showed higher antioxidant capability than the others while *Lippia citriodora* contained the highest amount of antioxidants.

Cyclic voltammetry is expected to be a simple method for screening antioxidants and estimating the antioxidant activity of foods and medicinal plants.

## Introduction

Oxidation is one of the most important chemical processes in food and chemicals. Free radicals can oxidize nucleic acids, proteins and lipids, initiating degenerative diseases (1-3).

Epidemiological evidence shows an association between decreased risk of cardiovascular disease and a diet rich in fruits and vegetables (4). Vegetables and fruits are also reported to decrease the risk of degenerative diseases and could have a protective effect against oxidative stress (5). These effects may be related to natural antioxidants, including phenolic acids, phytates, flavonoids, vitamin E and vitamin C which have the ability to scavenge free radicals and protect cells from the damage caused by free radicals (6-8). 

From this perspective food and plants become target samples for antioxidant screening. Several methods for evaluation of antioxidant activity (AA) of compounds have been developed, such as enzymatic or non enzymatic measurement of lipid peroxidation inhibiting effects, active oxygen species scavenging capability determination and radical scavenging activity determination including DPPH (1,1- diphenyl-2-picrylhydrasyl) radical (9-12).

It is clear that the data obtained by different assays reflect only the specific antioxidant capacity in the corresponding system. But it should be noted that antioxidant capacity is not dependent on one simple specific reaction and different mechanisms could be involved in antioxidant activity. In order to evaluate the overall antioxidant capability present in food and medicinal plants, electrochemical approaches without use of a certain reactive species could be applied (13).

Cyclic voltammetry is a unique technique for the electrochemical characterization of compounds by providing data about their oxidation/reduction potentials. Beside simplicity and rapidness, this technique is based on the chemico-physical properties of the molecules and can be widely used in evaluating antioxidant in oil and food stuff (14). The literature review shows that there is a good correlation between the oxidation potentials of various antioxidant and their antioxidant efficiency (15).

The cyclic voltammetry procedure reported by Kohen *et al.* (16) evaluated the overall reducing power of low molecular weight antioxidants in a biological fluid or tissue homogenate. Following preparation, the sample is introduced into a well in which three electrodes are placed: the working electrode (glassy carbon), the reference electrode (Ag/AgCl), and the auxiliary electrode (platinum wire). The potential is applied to the working electrode at a constant rate (100 mVs^-1^) either toward the positive potential (evaluation of reducing equivalent) or toward the negative potential (evaluation of oxidizing species). During operation of the cyclic voltmmmetry, a potential current curve is recorded (cyclic voltammogram). Recently quantitative determination of the phenolic antioxidants using voltammetric techniques was described by Raymundo *et al*. (17). In case of plant samples chatterjee *et al.* (18) determined the antioxidant property of green pepper and lignans from fresh mace. Kilmartin and Hsu (19) determined antioxidant property of green, oolong and black teas and in coffee using cyclic voltammetry method.

In this work, the antioxidant activities of some selected medicinal plants belonging to various subclasses were measured using cyclic voltammetry and compared with quercetin as a strong antioxidant.

## Experimental

*Chemicals*

All chemicals and reagents were obtained from Merck chemical company (Darmstadt, Germany) and were used without further purification.

*Plant materials*

The aerial parts of *Buxus hyrcana, Rumex crispus, Achillea millefolium, Zataria* *Multiflora, Ginkgo biloba, Lippia citriodora* and *Heptaptera anisoptera *were collected from Tehran province in july 2007. The plants were identified in Shahid Beheshti university by Faraz Mojab. A voucher specimen was deposited for each plant at the Herbarium of the Department of Pharmacognosy, Shahid Beheshti School of Pharmacy, Tehran, Iran.

*Extraction*

For each plant the fresh aerial parts were dried under shadow for 3 weeks. The powdered materials (100 g) were extracted three times with 300 mL methanol by maceration at room temperature overnight.

The solvent was filtered and evaporated under reduced pressure to afford a concentrated syrup.

**Figure 1:**
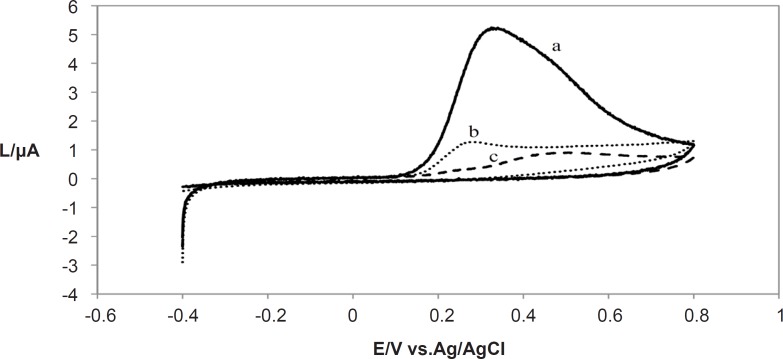
Overlapped Cyclic Voltammograms of methanolic extracts of *Lippia citriodora* (a) *Achillea millefolium *(b) and *Buxus hyrcana* (c) in pH 7.0 at a glassy carbon electrode (1.8 mm diameter); Scan rate: 10 mVs^-1^

*Determination of antioxidant activity by cyclic voltammetry*

Electrochemical measurements were carried out on a Metrohm VA 746 polarograph.

Cyclic voltammetric measurements were carried out using a conventional three electrode system in a single compartment cell. The three electrode system was composed of a glassy carbon working electrode (GCE), a saturated calomel reference electrode (SCE) and a platinum wire auxiliary electrode. Before each measurement the working electrode was polished with alumina on a polishing cloth. The plant extract solutions were prepared by dissolving the concentrated extract of each plant in methanol at concentration of 0.5 g/100 mL. Due to the low water solubility of some plant extract, acetonitrile was used in combination with water. Quercetin was used at concentration of 0.5 g/100 mL. A 0.2 M phosphate buffer pH 7.0 was used as the supporting electrolyte. Voltammetric signals were recorded at room temperature. All the experiments were performed in triplicates.

The cyclic voltammograms were recorded at scan rates of 10, 20, 30, 40, 50, 60, 70, 80, 90, 100, 200, 300 and 400 mVs^-1^. 

## Results and Discussion

The cyclic voltammograms for three different plant extracts shown in Figure 1 were recorded using a scan rate (*v*) of 10 mVs^-1^. Only one anodic peak appeared for them. No cathodic peak was observed on inverting the scan direction indicating the irreversibility of oxidation of the extracts of these plants at such a slow scan rate.

**Figure 2:**
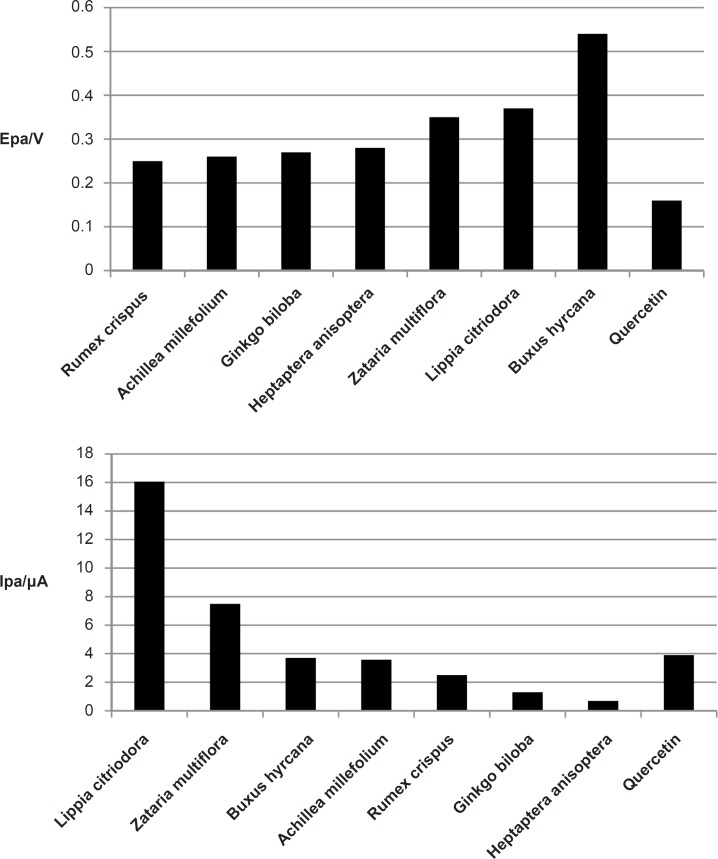
Comparison E_pa_ and I_pa_ values of medicinal plants at glassy carbon electrode.

All the extracts except the extract from *Buxus hyrcana *showed the peak anodic currents around +0.3V at scan rate of 100 mVs^-1^. The peak anodic current (I_pa _) was the highest for *Lippia citrodora* (16 μA) while it was minimum for *Heptaptera anisoptera* (0.7 μA). Quercetin as a standard antioxidant had peak anodic current at + 0.16 V and the amoumt of I_pa_ for this compound was 3.9 μA (Figure 2).

**Figure 3:**
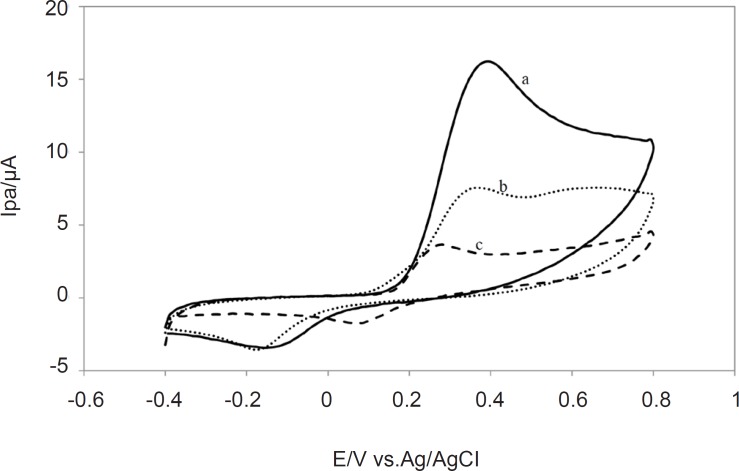
Overlapped Cyclic Voltammograms of methanolic extracts of (a) *Lippia citriodora* (b) *Zataria* *Multiflora* (c)* Achillea millefolium *in pH 7.0 at a glassy carbon electrode (1.8 mm diameter); Scan rate: 100 mVs^-1^.

The other aspect of voltammogram is that it shows the reversibility of the redox raction which takes place near the electrode. The peak anodic and peak cathodic currents will have equal magnitudes in a reversible process while for an irreversible reaction, the cathodic and anodic peaks are not equal and where the oxidation is very slow, no cathodic peak is observed. Therefore cyclic voltammetry could be used to characterize the redox behavior of plant extracts. The comparative voltammograms for the *Lippia citriodora, Zataria Multiflora *and* Achillea millefolium *and plant extracts is presented in Figure 3.

As it is shown in Figure 3, *Lippia citriodora *and* Zataria* *Multiflora *showed the highest I_pa.._ Since all the extracts were used at the same concentration of the total methanolic extract, higher I_pa _could be an indication of higher antioxidant content of these two extracts., On the other hand all the extracts except *Buxus hyrcana* showed the peak anodic currents in the vicinity of +0.3 V which means that they all have strong free radical scavenging capabilities and they are oxidized at relatively low potential. On the other hand *Buxus hyrcana* had E_pa _at +0.5 V which means it is less susceptible to be oxidized and hence has lower antioxidant (reducing) potential and it can exert prooxidant activity by autoxidation. 

In cyclic voltammetry when the potential becomes sufficiently less negative, the compounds near the electrode surface begins to be oxidized. This reaction gives rise to an anodic wave. Therefore the more susceptible the compound(s) is for oxidation, the sooner it will reach to its peak anodic current (E_pa_). On the other hand the magnitude of the peak anodic current (I_pa_) is proportional to the concentration of oxidizable species near the electrode which is in turn proportional to the concentration of the oxidizeable species in the bulk solution.

## Conclusions

The composition of food and medicinal plants are complex and it may be possible to have synergistic interactions among the antioxidant compound in plants material. Cyclic voltammetry can be used to characterize the antioxidant (reducing) ability of whole plant extracts. The measured oxidation potential could be closely related to the free radical scavenging capability of the investigated extract. By using the rapid and simple electrochemical methods without time consuming sample preparation, two parameters could be obtained: the anodic peak current (I_pa_) and the first oxidation potential (E_pa_). Low oxidation potentials in samples show their high antioxidant capacity. As many unknown structures could occur in the extract, since current is an additive magnitude, the amperometric current shows the contribution of all complex structures included in the extract (20).
